# FAS/FASL are dysregulated in chordoma and their loss-of-function impairs zebrafish notochord formation

**DOI:** 10.18632/oncotarget.2145

**Published:** 2014-07-01

**Authors:** Luca Ferrari, Anna Pistocchi, Laura Libera, Nicola Boari, Pietro Mortini, Gianfranco Bellipanni, Antonio Giordano, Franco Cotelli, Paola Riva

**Affiliations:** ^1^ Dipartimento di Biotecnologie Mediche e Medicina Traslazionale, Università Degli Studi di Milano, Via Viotti 3/5 20133 Milan, Italy; ^2^ Dipartimento di Bioscienze, Università Degli Studi di Milano, Via Celoria 26 20133 Milan, Italy; ^3^ Dipartimento di Neurochirurgia, Università Vita-Salute IRCCS Ospedale San Raffaele, Via Olgettina 60, 20132 Milan, Italy; ^4^ Department of Biology, College of Science and Technology, Temple University, Philadelphia, Pennsylvania 19122, USA; ^5^ Sbarro Institute for Cancer Research and Molecular Medicine, College of Science and Technology, Temple University, Philadelphia, Pennsylvania 19122, USA

**Keywords:** chordoma, FAS, FASL, notochord, zebrafish

## Abstract

Chordoma is a rare malignant tumor that recapitulates the notochord phenotype and is thought to derive from notochord remnants not correctly regressed during development. Apoptosis is necessary for the proper notochord development in vertebrates, and the apoptotic pathway mediated by Fas and Fasl has been demonstrated to be involved in notochord cells regression. This study was conducted to investigate the expression of *FAS/FASL* pathway in a cohort of skull base chordomas and to analyze the role of *fas/fasl* homologs in zebrafish notochord formation. *FAS/FASL* expression was found to be dysregulated in chordoma leading to inactivation of the downstream Caspases in the samples analyzed. Both *fas* and *fasl* were specifically expressed in zebrafish notochord sorted cells. *fas* and *fasl* loss-of-function mainly resulted in larvae with notochord multi-cell-layer jumps organization, larger vacuolated notochord cells, defects in the peri-notochordal sheath structure and in vertebral mineralization. Interestingly, we observed the persistent expression of *ntla* and *col2a1a*, the zebrafish homologs of the human *T* gene and *COL2A1* respectively, which are specifically up-regulated in chordoma. These results demonstrate for the first time the dysregulation of *FAS/FASL* in chordoma and their role in notochord formation in the zebrafish model, suggesting their possible implication in chordoma onset.

## INTRODUCTION

Chordoma is a rare slow-growing malignant tumor of notochordal origin. Chordoma can localize at Skull Base (SBC), sacral or spinal axis level, and accounts for approximately 0.1%–0.25% of intracranial tumors and 1%–4% of all malignant bone tumors [1, 2]. The treatment of choice for these tumors is en-bloc resection followed by postoperative radiation therapy [3]. To date chordoma is considered unresponsive to chemotherapy and no validated molecular markers are available to monitor the tumor progression [3]. Several line of evidences suggest that chordoma is characterized by abnormal regulation of notochord tissue. Histological studies have identified persistence of notochord tissue in this tumor that is localized along the axial skeleton, and expresses transcription factors that are also expressed in the notochord. Among them, the most significant is the transcription factor *T* (encoding for Brachyury), the founder member of the T-box family involved in notochord development [4-6], and recently identified as the pathognomonic marker for chordoma [7]. The genetic basis of *T* expression in chordoma is largely unknown [7-9], thus the question concerning the identification of early tumorigenic mechanisms leading to chordoma remains open and further pathways should be considered. Indeed, in a recent study other genes were found differentially expressed in both chordomas and related cell lines, among them the α1 collagen type II (*COL2A1*) was significantly over-expressed [10].

The proper balance between notochord cell proliferation and apoptosis seems to be fundamental for the development and regression of the notochord. The apoptotic process is involved in normal notochord development in *Xenopus laevis* [11], and in particular the extrinsic apoptotic pathway is conserved and necessary for notochord development in zebrafish [12]. In addition, the expression of tumor necrosis factor receptor (TNFR) *FAS* and its ligand *FASL*, leads to the notochord cells regression in the adult rat intervertebral disks [13, 14]. The autocrine-paracrine interaction between Fas and Fasl results in the trimerization and activation of the Fas receptor, which leads the cell to apoptosis [15, 16]. Multiple mechanisms regulate the sensitivity of Fas-expressing cells to Fas-induced apoptosis, including alternative splicing of *Fas* pre-mRNA: mRNAs lacking exon 6 encode soluble forms of the receptor which, sequestering Fasl, lead to a reduction of Fas signaling, inhibiting apoptosis [17]. Although molecular functions of Fas and Fasl are well known, their role during notochord development has never been investigated in full. Since *fas* and *fasl* showed conserved synteny between fish and mammals and also their functional domains are conserved [12], zebrafish (*Danio rerio*) represents a suitable animal model for functional studies.

The zebrafish notochord starts to form during gastrulation [18] and expresses *brachyury* (*ntl*) [19], sonic-hedgehog (*shh*) [20], and later *α1-collagen Type II* (*col2a1*) [21]. During the segmentation period, central cells of the notochord differentiate, acquiring a large vacuole and the notochord becomes surrounded by a sheath of tissue which, in combination with the turgor pressure generated by the vacuolated cells, imparts to the notochord its stiffness [22]. The differentiation correlates to apoptotic events that in the zebrafish happen between 14 and 24 hours post fertilization (14-24 hpf) [23]. As notochord cells become vacuolated, the expressions of *ntl*, *shh* and *col2a1* are extinguished in the notochord [19], while *shh* is maintained in the floor plate and *col2a1* in the floor plate and the perinotochordal sheath [21, 24].

Here we report the expression analysis of *FAS* and *FASL* and their downstream effectors Caspase 8 and Caspase 3 in a cohort of *SBC* samples, and functional studies of *fas* and *fasl* homologs genes during notochord formation in the zebrafish model. The obtained results indicate that *FAS/FASL* expression is dysregulated in chordoma and that the downstream Caspase 8 and 3 are mostly inactive in the *SBCs* analyzed. Moreover, simultaneous knock-down of *fas* and *fasl* in zebrafish resulted in defects during notochord formation and in vertebral mineralization. Interestingly, we also observed the maintenance of the expression of *ntla* and *col2a1a*, the zebrafish homologs of the human *T* gene and *COL2A1*, which were found to be specifically up-regulated in chordoma [10]. The obtained evidence strongly supports the implication of *FAS/FASL* in chordoma tumorigenesis.

## RESULTS

### FAS/FASL *pathway is inactivated in* SBCs

In our study we used *SBCs* samples that were validated at molecular level as bona fide *SBCs* samples. We also analyzed, by RT-PCR, the expression of the *T* gene in all the *SBCs* samples and U-CH1 chordoma cell line we used. In agreement with literature [10] *T* gene was expressed in all the samples. (Supplementary Material, Suppl. Fig. S1 A), while a pool of three *Nuclei Pulposi* (NP), commonly accepted as the reference control tissue for chordoma tumor since it is the only adult tissue of notochordal origin [25-28], was not expressing *T* gene. Western blot analysis, performed in a sub-group of twelve tumors and in U-CH1 cells, revealed the expression of Brachyury in all the samples analyzed, confirming the RT-PCR results (Suppl. Fig. S1 B) and the previous chordoma diagnosis obtained by immunohistochemistry (data not shown).

**Figure 1:**
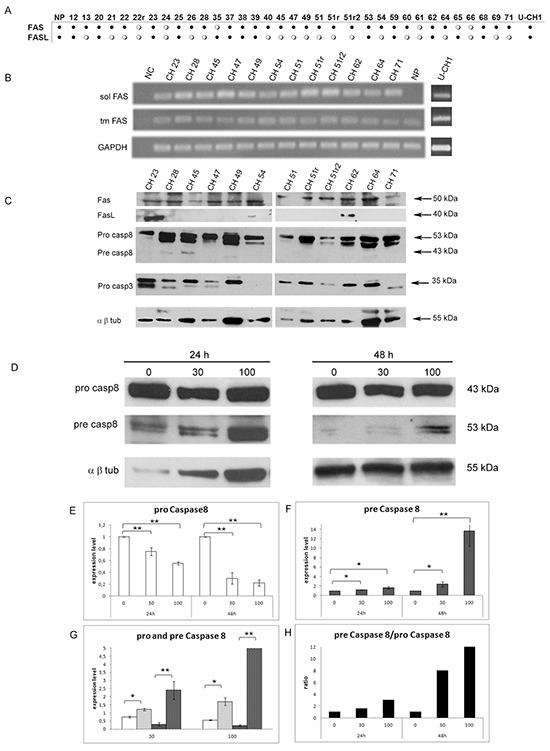
Expression analysis of *FAS*, *FASL* and effector Caspase 8 and 3 in a cohort of *SBCs* and U-CH1 cell line **(A)** RT-PCR results of *FAS* and *FASL* in 34 *SBCs*, *Nuclei Pulposi* (NP) and U-CH1 cell line; black dots indicate gene expression; white dots indicate no gene expression; **(B)** RT-PCR of antiapoptotic soluble *FAS* (sol FAS) and proapototic transmembrane *FAS* (tm FAS) in 12 *SBCs*, *Nuclei Pulposi* (NP) and U-CH1 cell line; NC indicates the RT-PCR negative control; **(C)** western blots of Fas, Fasl, Pro Caspase 8 (pro Casp8), Pre Caspase 8 (pre Casp8) and Pro Caspase 3 (pro Casp3) in 12 *SBCs* and U-CH1 cell line; the α β Tubulin (α β tub) was included as a housekeeping protein expression. **(D)** Western blot analysis of Pro caspase 8 (pro casp8), Pre caspase 8 (pre casp8) and α β Tub following U-CH1 cell line treatments with soluble FasL. U-CH1 were treated with soluble Fasl at the doses of 0 ng/mL (untreated control), 30 and 100 ng/mL for 24 and 48 hours, left and right panel respectively. **(E)** Quantification of relative Pro casp 8 expression levels after normalization to α β Tub in U-CH1 cells trated with Fasl, the white bars represent expression level of Pro casp 8. **(F)** Quantification of relative Pre casp 8 expression levels after normalization to α β tub in U-CH1 cells trated with Fasl, gray bars indicate the level of Pre casp 8. **(G)** Representation of the quantification of relative Pro casp 8 and of Pre casp 8 expression levels after normalization to α β Tub divided for each concentration, white bars indicate Pro casp 8 in U-CH1 cells treated with Fasl for 24 hours, white-dithered bars indicate Pre casp 8 treated with Fasl for 24 hours, gray bars indicate Pro casp 8 of Fasl for 48 hours, gray-dithered bars indicate Pre casp 8 treated with Fasl for 48 hours. **(H)** Ratios between Pre casp 8 and Pro casp 8 after treatments, the black bars represent the ratio for each treatment concentration at each time of exposure. **(D-G)** The data are expressed as fold increase over the untreated control (0 ng/mL); *p<0,05.

The transcription of *FAS* and *FASL* genes was studied by means of RT-PCR in the pull of NP, in thirty-four *SBCs* samples and in U-CH1 cells. Most of the analyzed samples (82%) showed *FAS* expression, while *FASL* transcript was present in only 38% of samples and U-CH1 cell line expressed both genes (Fig. 1 A). In order to determine the status of activation of Fas/Fasl apoptotic pathway in chordoma, we checked for the expression of the two different isoforms of *FAS*: the pro-apoptotic (transmembrane) form and the anti-apoptotic (soluble) form [29] by RT-PCR, in the sub-group of 12 tumors and in U-CH1 cells (Fig. 1 B). Interestingly, all the *SBCs* samples and the U-CH1 cells showed the expression of both, the transmembrane pro-apoptotic and the soluble anti-apoptotic, isoforms of *FAS*, while NP showed the solely expression of the pro-apoptotic transmembrane isoform (Fig. 1 B).

We also determined, in the same samples, the presence of Fas, Fasl Caspase 8 and Caspase 3 proteins by western blot analysis. We found the inactive Caspase 8 (pro Caspase 8) to be expressed in all the samples analyzed, while the active form (pre Caspase 8) was found weakly expressed only in 3 tumors (Fig. 1 C). The inactive form of pre Caspase 3 was detected in 83% of the samples (10/12 samples) (Fig. 1 C). These results suggest a specific role for the anti-apoptotic Fas in blocking the Caspase cascade and consequently the apoptotic pathway in chordoma samples and UCH1 cell line. To verify if the expression of anti-apoptotic Fas is responsible for this state, we exposed U-CH1 cells to synthetic soluble Fasl (SuperFAS Ligand), which is able to induce apoptosis by competing with the anti-apoptotic (soluble) isoform of Fas. The amount of Pro caspase 8 and of Pre Caspase 8 were evaluated by means of western blot analysis following soluble Fasl treatments at different concentrations and times of exposures. After Fasl treatments the levels of Pre caspase 8 significantly increased together with the significant decrease of Pro Caspase 8 levels in a dose and time exposure dependent manner (Fig. 1 D-G). Moreover, the ratio between Pre Caspase 8 and Pro Caspase 8 increased in a dose and time dependent manner (Fig. 1 H). These findings indicate that the administration of Fasl in U-CH1 cell line is able to counteract the role of the anti-apoptotic form of Fas and induces the apoptotic pathway via Pro Caspase 8, suggesting that this pathway can be modulated in this tumor.

### Zebrafish *fas* and *fasl* expression and function

**Figure 2:**
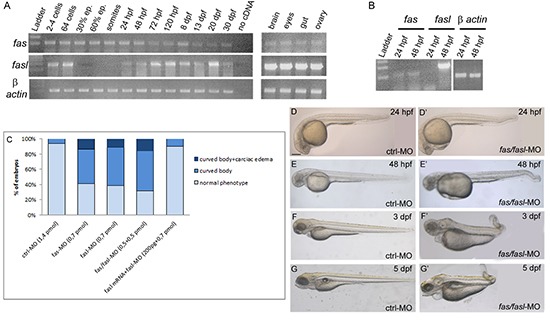
Expression and functional analysis of *fas* and *fasl* in zebrafish **(A)** RT-PCR performed on different developmental stages and adult tissues. *fas* is expressed in all the analyzed developmental stages, *fasl* presents a maternal expression, while the zygotic expression starts from 24 hpf. Both genes are expressed in all the adult tissues analyzed. **(B)** PCR performed on cDNA of the notochord cells sorted from embryos at 24 and 48 hpf injected with the *twhh*:*GFP/PCS2+* construct. *fas* is expressed in the notochord cells at both 24 and 48 hpf, while *fasl* only at 48 hpf. **(C)** Injected embryos for the single or double knock-down of *fas* and *fasl* and for the rescue of the phenotype, were analyzed by scoring the presence/absence of curved bodies and cardiac edema and subdivided into phenotypic classes. **(D-G)** Embryos and larvae injected with ctrl-MO exhibit normal development at 24 hpf **(D)**, 48 hpf **(E)**, 3 dpf **(F)** and 5 dpf **(G)**. **(D'-G')** Embryos and larvae co-injected with *fas*/*fasl*-MO develop defects in the tail curvature due to notochord distortion and cardiac edema. This phenotype is worsening during development.

Previous studies show contrasting results on the expression of *fas* and *fasl* in the zebrafish. Some reported that *fas* and *fasl* expression were not seen by whole-mount *in situ* hybridization (WISH) during the first stages of development [12], while in some cases their transcripts were detected by RT-PCR [12]. To clarify this point, we performed RT-PCR analyses of zebrafish *fas* and *fasl* in the whole developing embryo, larva and in some selected adult tissues (Fig. 2 A). We determined that *fas* is expressed in all the analyzed developmental stages, while *fasl* expression is modulated during development showing an initial maternal expression, *fasl* transcripts disappeared in the zygotic stages (30% epiboly-somites stages) to return at 24 hpf. To specifically analyze the expression of *fas* and *fasl* in the notochord, we labeled notochord cells by injected the pCS2+ (*twhh*:GFP) construct [30] and FACS-sorted GFP+ cells from embryos at 24 and 48 hpf, the GFP+ cells were then analyzed by RT-PCR. Expression of *fas* was detected in the notochord cells at both 24 and 48 hpf, while *fasl* was expressed only at 48 hpf (Fig. 2 B). Thus, like for the *SBCs*, in zebrafish embryogenesis the expression of *fas* is constant, while *fasl* transcription is modulated in time.

To investigate the function of *fas* and *fasl* in zebrafish development, we knocked-down these genes using oligonucleotide-antisense morpholinos (MO). As a control we injected a non-specific control MO (ctrl-MO). The single *fas*-MO and *fasl*-MO injection (0,7 pmol/embryo), or co-injection of *fas*/*fasl*-MO at a dosage that did not individually cause morphological defects (0,5 pmol/embryo/each) presented the same defective phenotypes. Therefore, for all the following results, we decided to present the data of the double knock-down (*fas*/*fasl*-MO) (Fig. 2 C). The *fas/fasl*-loss-of-function phenotype was characterized by bent notochord, curved tail and cephalic and cardiac edema (60% N=620, Fig. 2 D-G'), that was worsening during later stages of development. In addition, from 3 dpf, the most evident defect in *fas*/*fasl*-MO injected embryos was a high reduction in motility. The ctrl-MO injected larvae, when stimulated with the touch response assay, escaped in the opposite direction (100% N=50, Movie 1) while *fas*/*fasl*-MO injected larvae were characterized by an altered motility, swimming in circle (80% N=80, Movie 2). Following the injection of *fasl* mRNA in *fasl*-MO injected larvae, we were able to rescue the phenotype, confirming the specificity of the down-regulation (80% N=65, Movie 3). The *in-vivo* efficiency of the ATG morpholinos was tested with sensor plasmids (Supplementary Material, Suppl. Fig. S2 A-B', D-E'). Moreover, we designed splice-site morpholinos (splice-*fas*-MO and splice-*fasl*-MO; 1 pmol/embryo/each) that presented consistent phenotypes with the ATG morpholinos (80% altered motility N=400, 55% bent notochord, curved tail and cephalic and cardiac edema, N=320), confirming the specificity of the loss-of-functions. The efficiency of the splice-site-MOs was tested by means of RT-PCR (Supplementary Material, Suppl. Fig. S2 C,F).

### fas/fasl*-MO-injected embryos share similarities with chordoma tumors*

The most direct readout for the knock-down of the apoptotic genes *fas/fasl* is a decrease in apoptosis. Thus, we performed TUNEL assay at different developmental stages. To exclude undesirable off-target effects elicited by the injection of morpholino molecules, such as activation of the p53 protein [31], we injected *p53*-MO together with *fas/fasl*-MO and with control-MO. We observed that apoptosis was reduced in *fas/fasl/p53*-MO injected embryos compared to the control/*p53*-MO at the same developmental stages (50% N=60, Supplementary Material, Suppl. Fig. S3).

**Figure 3:**
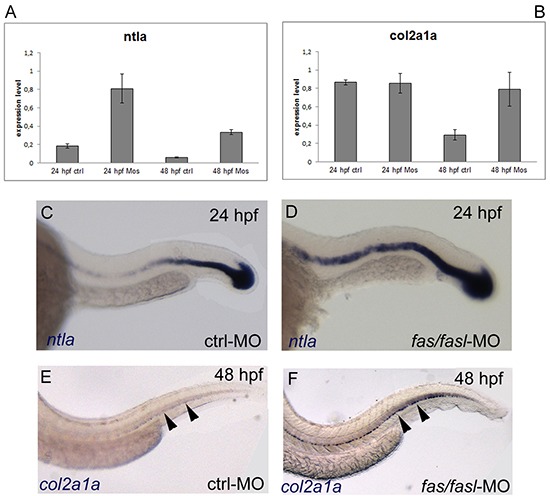
*ntla* and *col2a1a* are up-regulated in *fas/fasl*-MO injected embryos **(A-B)** q*-PCR* analysis of *ntla* **(A)** and *col2a1a* **(B)** show an upregulation of the expression in *fas/fasl-*MO injected embryos for both genes (*p≤0,05). **(C-D)** WISH analysis show persistent expression of the notochord marker *ntla* in the notochord of *fas/fasl*-MO injected embryos at 24 hpf **(D)** compared to control embryos **(C)**. **(E-F)** WISH analysis shows persistent expression of the chordamesoderm marker *col2a1a* in the peri-norochordal sheath of *fas/fasl*-MO injected embryos *(F*, arrowheads*)* compared to control embryos in which *col2a1a* expression normally decreases at 48 hpf (*E*, arrowheads).

Next, we sought to analyze the expression profile of the genes that have been found altered in the chordoma tumors, such as the *T* gene and *COL2A1*. Both the zebrafish homolog *ntla* and *col2a1a* were found to be significantly up-regulated in *fas/fasl-*MO-injected-embryos by qPCR analyses (p≤0,05) (Fig. 3 A-B). These results were confirmed by WISH analyses. The expression of *ntla*, that normally progressively decays from 20 hpf, was maintained at high levels in *fas/fasl*-MO injected embryos (50% of *fas/fasl*-MO-injected, total N= 30) (Fig. 3 C-D). Moreover, the expression of *col2a1a*, that normally diminished from 30 hpf and disappeared at 48 hpf [21, 32], persisted in the peri-notochordal sheath in *fas/fasl*-MO injected embryos at 48 hpf (70% of *fas/fasl*-MO-injected, total N= 60) (Fig. 3 E-F).

### Notochord architecture and surrounding tissues are affected in *fas/fasl*-MO injected larvae

**Figure 4:**
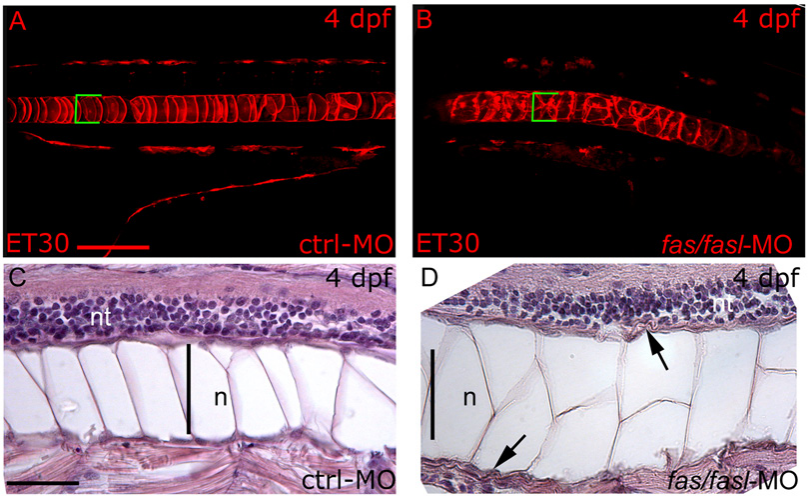
*fas* and *fasl* loss-of-function affects notochord differentiation and peri-notochordal sheath integrity **(A-B)** Confocal images of the mCherry-positive-notochord cells of the ET30 transgenic line at 4 dpf. In ctrl-MO injected larvae **(A)**, the notochord shows its characteristic “stack-of-coins” structure while *fas/fasl*-MO injected larvae **(B)** present notochord undulations and form multi-cell-layer jumps. The same region of the notochord has been analyzed in ctrl-MO and *fas/fasl*-MO injected larvae, as shown by the yolk extension. **(A-B)** Squared brackets indicate the diameter of the notochord. **(C-D)** Longitudinal sections hematoxilin-eosin (HE) stained of ctrl-MO and *fas/fasl*-MO injected larvae at 4 dpf. The notochord (n) of morphants is thicker, and vacuolated cells are not properly connected to the peri-notochordal sheat that is abnormally undulated (arrowheads, *D)*, in comparison to ctrl-MO injected larvae **(C)**. (*A-D)* lateral views, anterior to the left, dorsal up. Scale bars: **(A-B)** 100 μm, **(C-D)** 50 μm.

*fas* and *fasl* are expressed in the notochord, thus we analyzed possible defects caused by *fas/fasl*-loss-of-function in this structure and in the surrounding tissues. Taking advantage of the ET30: Et(kita:GalTA4,UAS:mCherry)hzm (ET30) transgenic line, where the fluorescent protein mCherry is expressed in notochord cells [33, 34], we were able to analyze the morphology of the notochord. Notwithstanding the curved tails and the notochord bents observed in *fas/fasl*-MO injected embryos starting from 24 hpf, no evident morphological defects in the notochord cells were shown before 48 hpf (data not shown). However, later during development (*i.e*. 4 dpf), the *fas/fasl*-MO injected larvae (80%, N=70) presented notochord undulations and multi-cell-layer jumps (Fig. 4 B) instead of the characteristic single “stack-of-coins” structure seen on ctrl-MO injected larvae (N=70) (Fig. 4 A). Moreover, longitudinal sections of *fas/fasl*-MO injected larvae at 4 dpf showed that the entire notochord structure was bigger in comparison to ctrl-MO injected larvae and the larger vacuolated cells were not properly connected to the peri-notochordal sheath, indicative of a failure of the cells to differentiate (Fig. 4 C-D). Indeed, the peri-notochordal basement membrane of *fas/fasl*-MO injected larvae at 4 dpf, was abnormally undulated and thicker than the ctrl-MO injected larvae, in particular in areas where the profile of the notochord is bent (Fig. 4 C-D). The phenotypic defects in notochord structure are specifically caused by *fas/fasl*-loss-of-function as rescued larvae (75% N=120) did not present such defects (Supplementary material, Suppl. Fig. S4 A-C). We considered the possibility that the defects in notochord morphology might have resulted from a general developmental delay, although *fas/fasl*-loss-of-function embryos did not differ noticeably in overall development from ctrl-MO. To verify this hypothesis we compared the vessels formation in ctrl-MO and *fas/fasl*-MO injected larvae using Tg(*flk1:*EGFP) fish line. In both cases, vessels formation at 4 dpf was comparable, (control N=40; *fas/fasl*-MO N=40) [35], as shown in the Suppl. Fig. S5, indicating that both ctrl-MO and *fas/fasl*-MO injected larvae were at the same developmental stage.

### Muscle organization and primary motoneuron axonal projections are altered in *fas/fasl*-MO injected larvae

**Figure 5:**
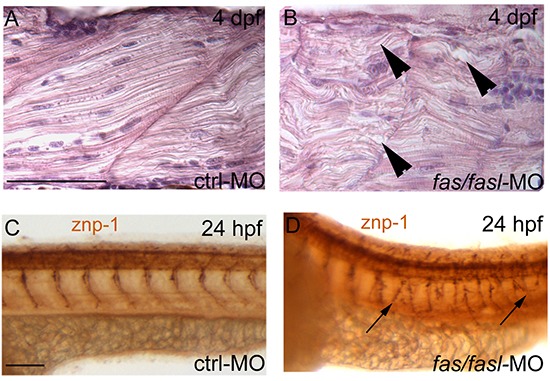
Defects in notochord differentiation prevent normal muscle structure and primary motoneuron axon projections **(A-B)** Longitudinal histological sections, HE stained. At 4 dpf, muscle fibres in *fas/fasl*-MO injected larvae are disorganized, undulated and oriented in opposite directions **(B, arrowheads)** in comparison to ctrl-MO injected larvae **(A)**. **(C-D)** Axonal projections of primary motoneurons visualized by znp1 antibody present branching defects in *fas/fasl*-MO injected embryos at 24 hpf (arrows). **(A-D)** lateral views, anterior to the left, dorsal up. Scale bar: 100 μm.

The impaired motility of *fas/fasl-*MO injected larvae from 3 dpf, prompted us to analyze the muscle structure by means of histological sections: at 4 dpf *fas*/*fasl*-MO injected larvae showed muscles with a disorganized alignment of myofibrils that appeared undulated and unusually oriented (80% N=40) compared to the controls (N=40) (Fig. 5 A-B). Motility impairment could be due to motoneuron defects, thus we analyzed primary motoneurons and their axon (visualized by the znp1 antibody); they formed in a proper number and position in *fas/fasl*-MO injected embryos (N=30) at 24 hpf compared to the controls (N=30). However, the disorganization in muscle and myosepta caused a disorganized branching of axonal projections (Fig. 5 C-D). Rescued larvae (89% N=170 for muscle defects, Supplementary material, Suppl. Fig. S4 D-F; 75% N=80 for motoneuron defects, Supplementary material, Suppl. Fig. S4 G-I) did not present such defects.

### Notochord defects in *fas/fasl*-MO injected larvae lead to abnormal vertebral development

**Figure 6:**
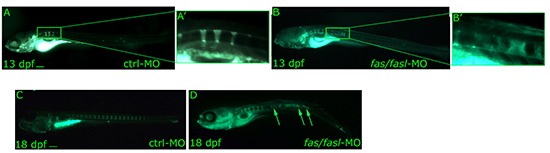
Analyses of notochord segmentation and vertebral formation following *fas/fasl* loss of function **(A-D)** Calcein staining shows notochord segmentation by formation of calcified chordacentra in an antero-posterior fashion. The process of vertebrae formation starts at around 11 dpf (3 mm) and is completed at around 18-21 dpf (7-9 mm). *fas/fasl*-MO-injected larvae at early **(B-B'** higher magnification*)* and complete vertebral mineralization **(D)** show significant defects in vertebrae formation with extensive vertebrae fusion (**arrows, D**) in comparison to ctrl-MO injected larvae **(A, A'** higher magnification, **C**). Scale bar: 100 μm.

Several evidences suggest that the notochord has been directly implicated in the formation of vertebrae and intervertebral discs [36, 37]. Therefore, we verified whether defects in notochord differentiation in *fas/fasl-*MO injected larvae could influence subsequent vertebral formation. We calcein stained *fas/fasl*-MO injected larvae at early (13 dpf, around 5 mm, Fig. 6 A-B') and complete vertebral mineralization (18 dpf, around 7-9 mm, Fig. 6 C-E) and we showed defects in vertebrae formation with extensive vertebrae fusion (15% N=30, Fig. 6 B,B',D) in comparison to ctrl-MO injected larvae (0% N=30, Fig. 6 A,A',C). Also the defects in vertebrae mineralization are specifically caused by *fas/fasl*-loss-of-function as the majority of rescued larvae did not present such defects (79% N=28), (Supplementary material, Suppl. Fig. S4 J-L).

## DISCUSSION

Chordoma is a rare malignant tumor that is thought to arise from notochord remnants. The notochord regression is regulated by several mechanisms, and among them, the apoptotic process plays a relevant role [11, 38, 39]. In particular, the Fas/Fasl pathway was implicated in the regression of the notochord cells during adult *nucleus pulposus* formation in rat [13]. In this work, we demonstrated for the first time that *FAS/FASL* expression was dysregulated in chordoma, mainly for the absence of *FASL*. Furthermore, all *SBC* specimens and the U-CH1 cell line, including those expressing *FASL*, showed the expression of the *FAS* soluble anti-apoptotic isoform that was not detected in the reference tissue of NP. It is known that the soluble anti-apoptotic form of Fas competes with the transmembrane pro-apoptotic form in binding FASL and therefore inhibits apoptosis [17]. The prevalent expression of the inactive form of the downstream effectors Caspase 8 and Caspase 3 confirmed the inactivation status of the related apoptotic pathway in the *SBCs* analyzed. We were able to revert this status in U-CH1 cells by exposition to synthetic soluble Fasl, activating Caspase 8 in a time and dose dependent manner, showing that, *in vitro* chordoma cell line, the *FAS/FASL* pathway can be modulated.

The expression studies performed in our cohort of *SBCs* suggest a possible involvement of *FAS* and *FASL* in chordoma onset. However, from *in vitro* and chordoma samples we could not determine if this genetic expression profile is the cause or the result of the *SBC* [7]. Aiming to elucidate this ontological question of cancer etiology, we began to investigate the effects of the dysregulation of *FAS/FASL* in notochord during embryo development. We performed functional assays in the zebrafish model. We firstly evaluated the expression of *fas* and *fasl* in zebrafish whole embryos and larvae. The expression of *fas* and *fasl* in brain, eyes, gut and ovary of the adult fish is conserved in mammals, indicating a similar role for *FAS/FASL* during evolution [14, 40-43]. Interestingly, *fasl* expression is modulated during development while *fas* is expressed in all stages analyzed, suggesting the importance of a specific activation of this factor. Moreover, we detected *fas* and *fasl* expression in the zebrafish notochord sorted cells, pinpointing for the first time the involvement of these two genes in the processes of notochord formation.

By using morpholino technology, we performed loss-of-function experiments to analyze notochord defects in zebrafish embryos and larvae. As expected, a reduction of apoptosis was observed in *fas/fasl*-MO injected embryos. In addition, we found a significant up-regulation of two homologs of chordoma markers, *ntla* (*T*) and *col2a1a* (*COL2A1*) [10]. The maintained expression of these two genes in *fas/fasl-*MO-injected embryos in a developmental stage in which they normally diminished and disappeared, might suggest molecular alteration common to chordoma. The up-regulation of the *col2a1a*, as well as deregulation of other genes expressed in the notochord or in the perinotochordal sheath, such as *col15a1, col27a1a* and *col27a*, are linked to defects of the notochord sheath and aggregation of protein in notochord cells, as demonstrated in previous reports [44, 45]. Consistently with these works, we found severe alterations of the notochord morphology that presented various degrees of packed cells that were larger and not properly connected to the perinotochordal sheath. In addition, the loss-of-function of *fas/fasl* produced disorganized myofibrils and an aberrant primary motoneurons branching, resulting in motility impairment. Indeed, both muscle and motoneuron formation require proper signaling from the notochord, and it has been demonstrated that also the integrity of the perinotochordal sheath is essential for the axon projections [46-49].

Transient depletion of *fas*/*fasl* resulted, later in development, in vertebrae mineralization defects instead of the normal notochord ossification [32]. *fas/fasl* loss-of function might alter the proper notochord cells disappearance during notochord regression, similarly to what happens to the notochord cells in the nucleus pulposus of rat [13]. This might cause the mechanical weakening of notochord sheath leading to defects in vertebrae formation [48]. It is worth to note, that all the phenotypic defects we observed are specifically caused by a transient *fas/fasl*-loss-of-function [59]. Our data show how transient deregulation of this pathway during embryo development can cause a cascade of effects seen much later in the larvae and reminiscent of some of chordoma characteristics. However, due to the transient condition of our loss-of-function we did not obtained a chordoma-like tumor in the zebrafish.

In a recent work, a zebrafish model of chordoma has been obtained constitutively expressing the HRAS pathway [50].

The upregulation of *T* gene, involved in the regulation of cell cycle control, might lead to notochord cells proliferation [5, 51]; in alternative we speculated that the defects in notochord regression, through apoptosis alterations, may maintain proliferating notochord cells expressing the *T* gene, or both these possibilities [7]. Neverteless, the evidence here provided indicates the early role of *FAS/FASL* pathway during embryogenesis as a possible cause of chordoma formation [57, 58], and suggests the need for persistent dysregulation of *FAS/FASL* pathway in later stages as a determinant for tumor onset. Several mechanisms such as methylation and/or post-transcriptional expression modulation by specific miRNAs, control the expression of *FAS* and *FASL* [52-54]. The dysregulation of one or more of those mechanisms during development and later in chordoma might be the cause of *FAS/FASL* altered expression. In turn, a dysregulation of *FAS* alternative splicing is caused also by the enhanced expression of the anti-apoptotic *FAS* isoform in chordoma [29, 55, 56].

This study provides new insights on notochord biology and indicates the implication of Fas/Fasl in chordoma, addressing future areas of investigation to identify new targets for chordoma treatment and diagnosis.

## MATERIAL AND METHODS

### Patients

The cohort includes fourteen *SBCs* patients described for the first time in this study and seventeen patients previously reported [25], for a total of thirty-one patients with *SBC* (Table 1), each of whom underwent surgery at the Department of Neurosurgery of the San Raffaele Hospital in Milan, Italy, between August 1997 and December 2011. Twenty-three patients were males (71.9%), six were females (28.1%); ages ranged from 19 to 71 years (average 47, 7 years; SD = 15.24). Six patients (19%) had been treated previously. The histological specimens were reviewed in each case by the same pathologist for the presence of specific immunohistochemical markers, diagnostic of chordoma (S-100, vimentin, EMA, cytokeratine). The *Nuclei Pulposi* (NP) were obtained after surgical excision of intervetebral herniated disks from three young individuals. The expression study carried out on the surgical specimens did not impact neither the course of surgical operations nor the decision about post-operative adjuvant therapies. Informed consent for molecular genetic researches on the surgical specimens was signed by all the patients before operations.

**Table 1:** Cohort of patients

Patient	Sex	Age	Histology	Recurrence*	Death*	Description
12	M	67	chondroid	-	-	Riva et al. 2003
13	M	55	classic	-	-	Riva et al. 2003
20	M	55	classic	41	47	Riva et al. 2003
21	M	46	classic	-	-	Riva et al. 2003
22	M	40	classic	22	30	Riva et al. 2003
23	M	56	-	-	-	Riva et al. 2003
24	F	29	chondroid	-	-	Riva et al. 2003
25	M	27	chondroid	24	53	Riva et al. 2003
26	M	52	classic	-	-	Longoni et al. 2008
28	F	69	-	-	-	Longoni et al. 2008
35	F	25	classic	-	-	Longoni et al. 2008
37	F	41	classic	-	-	Longoni et al. 2008
38	M	31	classic	-	-	Longoni et al. 2008
39	F	32	chondroid	-	-	Longoni et al. 2008
40	F	30	chondroid	16	-	Longoni et al. 2008
45	M	52	chondroid	5	7	Longoni et al. 2008
47	M	66	-	-	-	This work
49	M	46	classic	-	-	Longoni et al. 2008
51	M	55	classic	36	-	This work
53	M	47	chondroid	-	-	This work
54	M	nd	-	-	-	This work
59	F	69	chondroid	-	-	This work
60	F	61	classica	-	0	This work
61	M	65	classica	-	-	This work
62	M	71	classic	-	-	This work
64	M	46	classic	-	-	This work
65	M	49	classic	-	-	This work
66	M	45	-	-	-	This work
68	M	20	-	-	-	This work
69	M	62	-	-	-	This work
71	M	19	classic	-	-	This work

**Abbreviations:** M, male; F, female;

* months after surgery

### U-CH1 chordoma cell line and treatments

Chordoma cell line U-CH1 was obtained from the Chordoma Foundation and it was maintained in IMDM (Invitrogen 12440) / RPMI1640 (Sigma-Aldrich Milan, Italy) four to one ratio (4:1), supplemented with 10% FBS (EuroClone, Milan, Italy) and 100 u/mL penicillin/streptomycin (Sigma-Aldrich) at 37°C and 5% CO_2_ [10]. The cells were seeded in coated plates or flasks (Collagen Cellware Becton DickinsonSan Jose, CA) and treated with SuperFAS Ligand (soluble Fasl, Enzo Life Science, Farmingdale, NY) at different times and concentrations. SuperFAS Ligand was reconstituted with 50 μL sterile water to 0.1 mg/mL and stored at −20°C, according to the manufacturer's instructions.

### Animals

Breeding zebrafish was maintained at 28°C on a 14 h light/10 h dark cycle. Embryos were collected by natural spawning, staged and raised according to Kimmel and colleagues [18], in agreement with EU Directive 2010/63/EU for animal. We express the embryonic ages in somites (s), hours post fertilization (hpf) and days post fertilization (dpf). Fish lines were described in Supplementary Materials.

### RT-PCR

Total RNA from tumor samples was extracted from frozen samples by using Trizol reagent (Life Technologies, Carlsbad, CA, USA) according to the producer's instructions. Reverse transcription (RT) was carried out on 1 μg of total RNA using the iScript™ cDNA Synthesis kit (Bio-Rad Laboratories Inc. Barkeley, CA, USA). The specific PCR primers for *FAS* isoforms were already reported [17]. *FASL* and *T* specific primers are reported in Supplementary Table 1. Total RNA from 17 zebrafish samples (an average of 30 embryos/larvae per sample) was extracted with the TOTALLY RNA isolation kit (Ambion, Life Technologies, Paisley UK), treated with RQ1 RNase-Free DNase (Promega, Madison WI, USA) and reverse transcribed using SuperScript II RT (Life Technologies, Carlsbad CA, USA). Primers list in Supplementary Table 1.

### RT and quantitative real time PCR (qPCR)

RTs were performed using 1 μg of DNase treated (DNA-*free*™, Ambion) total RNA in presence of random hexamers (Life Technologies) and SuperScript II reverse transcriptase (Life Technologies). qPCRs were carried out as reported in Malafoglia [57] and colleagues and also Supplementary Materials.

### Western blot analysis

The whole proteins extraction was performed on fresh/frozen specimens from the new enrolled patients, which were disaggregated into a SDS-PAGE sample buffer containing protease inhibitors as in Bellipanni and colleague [58]. The antibodies (Ab) used and specific dilutions are reported in Supplementary Materials.

### *In situ* hybridization, histological analysis and immunohistochemistry

Whole mount *in situ* hybridization (WISH) experiments, were carried out as described by Thisse and colleagues [59]. Immunohystochemistry was carried out as described in Panzer and colleagues [60]. Probes and antibodies were described in Supplementary Materials. For histological sections, embryos/larvae were re-fixed in 4% PFA, dehydrated and stored in methanol, wax embedded, sectioned (5-8 μm) and Hematoxilin/Eosin (H/E) stained. Images acquirement was described in Supplementary Materials.

### TUNEL staining

For TUNEL assay, a minimum of 24 embryos per treatment were fixed with 4% PFA for 2 h at room temperature. Embryos were washed with methanol at −20°C and then twice with PBC (0.001% Triton X-100, 0.1% sodium citrate in PBS) for 10 minutes. Staining for apoptotic cells was performed using the In situ Cell Death Detection Kit (Roche) according to manufacturers' instructions.

### Injections

Injections were carried out on 1- to 2-cell stage embryos; the dye tracer rhodamine dextran was also co-injected. The PCS2+ plasmid containing *twhh:*GFP construct was kindly provided by J. Du [37] and injected at a concentration of 200 pg/embryo. Morpholino description and validation was performed as described in Supplementary Materials. Morpholino sequences list in Supplementary Table 2.

### Sorting

70-100 embryos at 24 and 48 hpf injected with the pCS2+ (*twhh*:GFP) construct, were dissociated and GFP*+* cells were sorted using a Vantage Sorter SE (Becton-Dickinson, San Jose CA, USA) at a flow rate of 3000 cells per second. GFP was exited at 488 nm using an argon laser. Cells dissociated from wild-type embryos were used to set the gating to exclude green autofluorescence. RNA was extracted from sorted cells with the micro-RNAeasy kit (Qiagen, Venlo, Netherlands), retro-transcribed with the iSCRIPTtm cDNA synthesis kit (Biorad).

### Confocal images

Live ET30:Et(kita:GalTA4,UAS:mCherry)hzm transgenic fish were anesthetized in a 0, 5% tricaine solution in fish water, mounted in a 1% low melt agarose and imaged on a Leica TCS NT confocal microscope.

### Calcein staining

Calcein (Sigma-Aldrich, St. Louis MO, USA) staining was done according to Du and colleagues [30, 37].

### Statistical analysis

For qPCR experiments, data were statistically analyzed applying a two-tailed T-test setting p≤0.05 as significant. Data were analyzed using the comparative ΔΔCt method both t test and SD values refer to samples triplicates.

## SUPPLEMENTARY MATERIALS, FIGURES AND TABLE


