# Supplementary material for: FAS/FASL are dysregulated in chordoma and their loss-of-function impairs zebrafish notochord formation
Source: Oncotarget. 2014 Jul 1;5(14):5712–24. doi: 10.18632/oncotarget.2145 (PMC4170636; doi:10.18632/oncotarget.2145)
Supplement: Supplementary file 1 [file oncotarget-05-5712-s001.pdf]

# SUPPLEMENTARY MATERIALS

## *Zebrafish lines*

The following zebrafish lines were used: AB from the S. Wilson lab, UCL, UK, the ET30:Et(kita:GalTA4,UAS:mCherry)hzm line, kindly provided by R. W. Koster and M. Mione [1, 2], IFOM, Milan Italy, and Tg(*flk1*:EGFP) obtained from the Lawson lab, University of Massachusetts, Boston, USA.

## *qPCR*

qPCR on zebrafish embyos (minimum 30 per experimental group) were carried out in a total volume of 25 µl containing half volume of SYBR Green (Life Technologies) qPCRs were carried in a total volume of 15 µl containing 1X iQ SYBR Green Super Mix (BioRad, Barckley CA, USA), using 1 µl of the RT reaction. PCRs were performed using the BioRad iCycler iQ Real Time Detection System (BioRad). For normalization purposes, *ef1-alpha* RNA level was tested in parallel with the gene of interest. Primers list in Supplementary Table 1.

## *Western blot analysis*

Protein samples were separated by 12% SDS-PAGE, Western blotted onto nitrocellulose membrane (Whatman protran BA 85, 0.45 µm) (ImmobilonTM-P, Millipore, Billerica, MA, USA). Following a blocking with 5% BSA in Tris-buffered saline, the membranes have been probed with the following antibodies (Ab): diluted 1:1000 p53 (Bp53-12) human anti-mouse monoclonal IgG (Santa Cruz Biotechnology, Santa Cruz, CA, USA); diluted 1:1000 Brachyury (N-19) rabbit polyclonal IgG (Santa Cruz Biotechnology); diluted 1:1000 Fas (C18C12) human anti-rabbit monoclonal IgG (Cell Signaling Technology, Beverly, MA, USA), diluted 1:1000 FASL rabbit policlonal IgG (Cell Signaling Technology); diluted 1:1000 Caspase 8 (1C12) human anti-mouse monoclonal IgG (Cell Signaling Technology); diluted 1:1000 Caspase 3 (8G10) human anti-rabbit monoclonal IgG (Cell Signaling Technology); diluted 1:2000 αβ Tubulin anti-rabbit policlonal IgG (Cell Signaling Technology), diluted 1:10000 mouse anti-β-tubulin (Covance). The incubation with secondary antibodies for 1 h at room temperature have been performed using the following antibodies: diluted 1:2000 ECL-rabbit IgG, HRP (horseradish peroxidise)-linked whole Ab from donkey (Amersham, Piscataway, NJ, USA) or 1:1000 or goat anti-mouse IgG-HRP (Santa Cruz Biotechnology); diluted 1:40000 HRP goat anti-rabbit IgG (Pierce, Rockford, IL, USA) or diluted 1:20000 HRP goat anti-mouse IgG (Sigma-Aldrich).

## *In situ hybridizations and immunohistochemistry*

*col2a1a* probe has been kindly provided by J. Topczewski laboratory and *ntla* probe was cloned as already reported [3]. znp1 antibody (mouse anti-*syt2b*) was purchased from Zebrafish International Resource Center (ZIRC). Secondary antibody was EnVision+ System- HRP Labelled Polymer anti-mouse (Dako, Glostrup, Denmark).

## *Images acquirements*

Images of embryos and sections were acquired using a microscope equipped with a digital camera with LAS Leica imaging software (Leica, Wetzlar, Germany). Images were processed using the Adobe Photoshop software and when necessary, different focal images planes of the same image have been took separately and later merged in a single image.

## *Loss-of-function experiments in zebrafish*

To repress *fasl* and *fas* mRNA translation, an ATG-targeting morpholino (*fas*-MO, *fasl*-MO) and a splice-MO were synthesized (splice-*fas*-MO, splice-*fasl*-MO) (Gene Tools LLC, Philomath OR, USA) and used at the concentration of 0,5 or 0,7 pmol/embryo in 1x Danieau buffer (pH 7,6) as previously reported [4]. As control, we injected a standard control morpholino oligonucleotide (ctrl-MO, Gene Tools LLC). The *p53* morpholino has been designed (GeneTools) and used as described previously [5].

## *Validation of fas/fasl- ATG- and splice site- morpholino oligos:*

The *in-vivo* test of the specificity was carried out as described in Brusegan and colleagues [6]. Sensor plasmids containing the sequence targeted by *fasl* (pCS2/*fasl*-MO-EGFP) and *fas* morpholinos (pCS2/*fas*-MO-EGFP) in frame with the EGFP sequence, were co-injected with *fasl*-MO, *fas*-MO or ctrl-MO respectively (Suppl. Fig. S2). The presence/absence of the EGFP was monitored at 24 hpf. Most (80% N=25) of the embryos injected with the sensor plasmid and the ctrl-MO were positive for the EGFP (Suppl. Fig. S1 A-A', D-D'). This percentage decreased to 15% (N=50) when the plasmids were co-injected with *fasl*-MO (Suppl. Fig. S2 B-B') or *fas*-MO (Suppl. Fig. S2 E-E'), indicating that morpholinos specifically bind to their target regions.

For the specificity of phenotype, 0,7 pmol of *fasl*-MO were injected together with 200 pg/embryo of endogenous *fasl* full-length mRNA. Synthetic capped *fasl*

mRNA was cloned into the PCS2+-expression-vector and transcribed with the mMessage kit (Ambion). Primers list in Supplementary Table 1.

RT-PCR experiments were done to validate splice-site-MO (splice-*fasl*-MO and splice-*fas*-MO) and verify intron retention or exon skipping respectively. Primers list in Supplementary Table 1. RNA was extracted from embryos at 48 hpf injected with the splice-site-MO. RT-PCRs were performed as previously described and showed abnormal splicing in embryos injected with *fasl* (Suppl. Fig. S2 C) and *fas* respectively (Suppl. Fig. S2 F), and normal splicing in control-MO injected embryos.

## SUPPLEMENTARY FIGURES AND TABLE

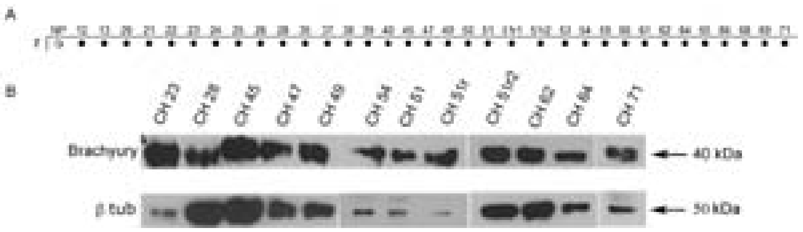


**Supplementary Figure S1: *T*/Brachyury expression in *SBCs*.** **(A)** RT-PCR results of *T* gene expression in 34 *SBCs*, and in nucleus pulposus (NP), black dots indicate gene expression; white dots indicate no gene expression; **(B)** western blots of Brachyury in 12 SBCs, the β Tub was included as a housekeeping protein expression.

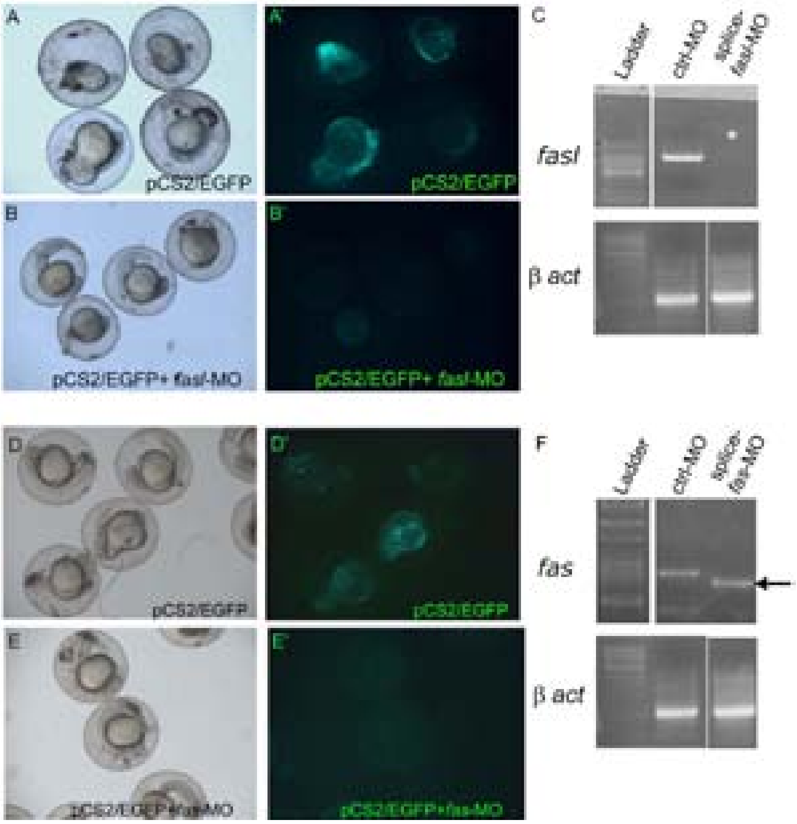


**Supplementary Figure S2: Validation and specificity of *fas*- and *fasl*-MO.** For the *in-vivo* test of the specificity of *fasl*-MO, a *fasl*-EGFP sensor has been generated. The pCS2+ construct containing the sequence recognized by the *fasl*-MO fused with the EGFP open reading frame is used for injection experiments with ctrl-MO or with the *fasl*-MO. **(A-A')** Embryos at 24 hpf: EGFP-positive cells in the trunk and in the yolk epithelium following co-injection of the sensor and the control-MO. **(B-B')** The complete absence of EGFP expression when the sensor is co-injected with *fasl*-MO confirms the specificity of the ATG targeting morpholino action. **(D-E')** Same experiments have been performed to *in-vivo* test the specificity of the *fas*-MO. In *A,B,D,E* embryos are visualized under normal light, in **A',B',D',E'** under fluorescent light. **(C)** RT-PCR on control and splice-*fasl*-MO injected embryos at 24 hpf. RT-PCR primers are designed in exon 1 and exon 2 respectively; the amplification product is present in ctrl-MO injected embryos, while it is not detected (asterisk) in splice-*fasl*-MO injected embryos, confirming the intron retention. **(F)** RT-PCR performed on control and splice-*fas*-MO injected embryos at 24 hpf. RT-PCR primers are designed in exon 1 and exon 3 respectively. The amplification product, that comprehends the second exon, is 326 bp in ctrl-MO injected embryos, while a band at 174 bp (arrowhead) is detected in splice-*fas*-MO injected embryos, confirming the skipping of the second exon (150 bp). Ladder is 1000 bp for *fasl* and *beta actin* PCR reactions, 100 bp for *fas* PCR reaction.

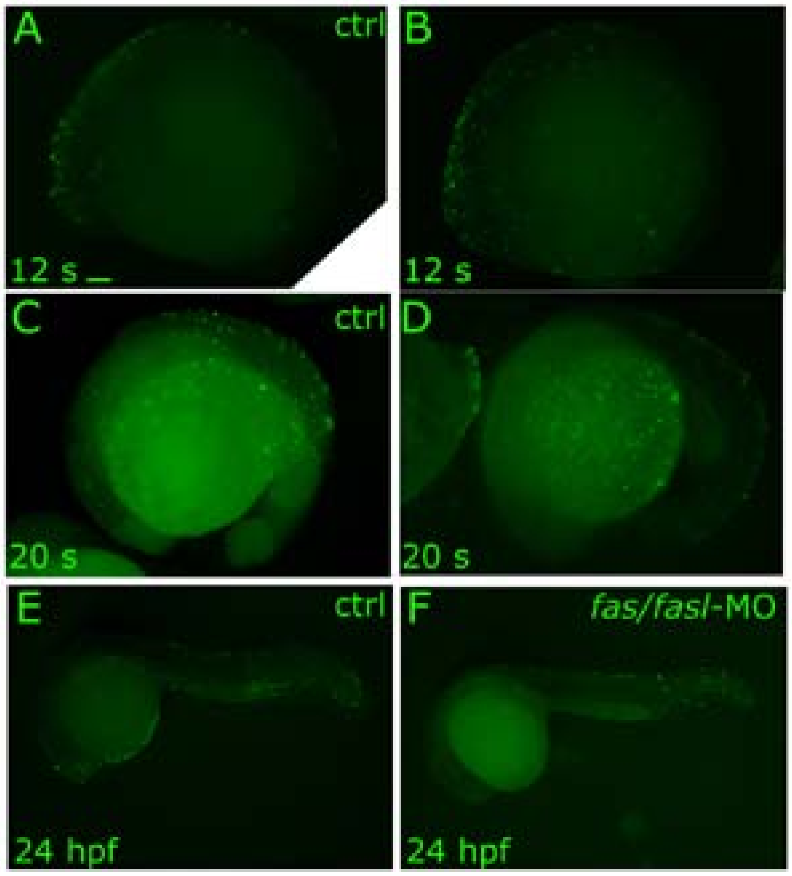


**Supplementary Figure S3: Apoptosis is reduced in *fas*/*fasl*/*p53*-MO injected embryos.** **(A-F)** TUNEL staining performed on whole embryos at different developmental stages. Apoptosis is reduced in *fas*/*fasl*/*p53*-MO injected embryos **(B, D, F)** compared to the control/*p53*-MO injected embryos at the same developmental stages **(A, C, E)**. Scale bar: 100 µm.

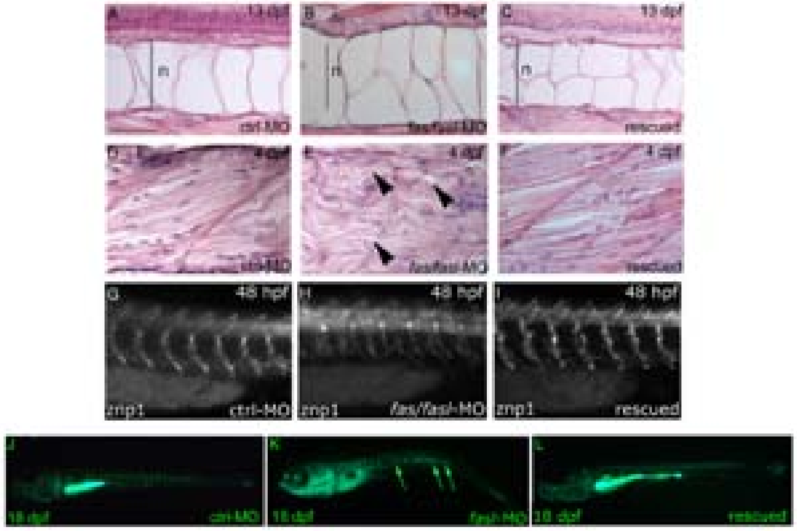


**Supplementary Figure S4: Rescued larvae recover the phenotypical defects caused by *fas/fasl*-downregulation.** The phenotypic defects in notochord structure **(A-C)**, muscle organization **(D-F)**, motoneuron axonal projections **(G-I)**, and vertebrae mineralization **(J-L)** are specifically caused by *fas/fasl*-loss-of-function as the majority of rescued larvae did not present such defects. **(A-L)** Lateral views, anterior to the left, dorsal up. Scale bars: **(A,B,C,D,E,F)** 50 µm; **(G,H,I,J,K,L)** 100 µm.

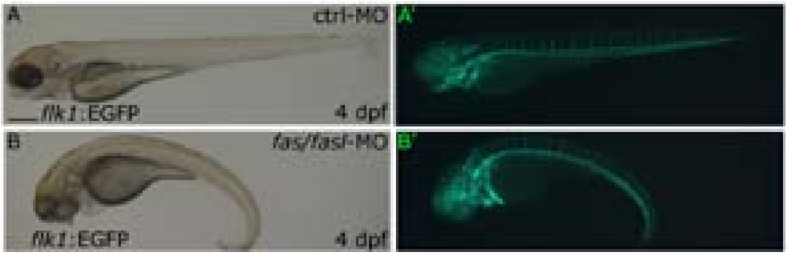


**Supplementary Figure S5: Vessel formation is comparable in control and *fas/fasl*-MO *flkI:*EGFP-transgenic injected embryos.** Visible **(A-B)** and fluorescent images **(A'-B')** of EGFP-vessels visualized in control **(A-A')** and *fas/fasl*-MO **(B-B')** injected embryos at 4 dpf. Scale bar: 100 µm.

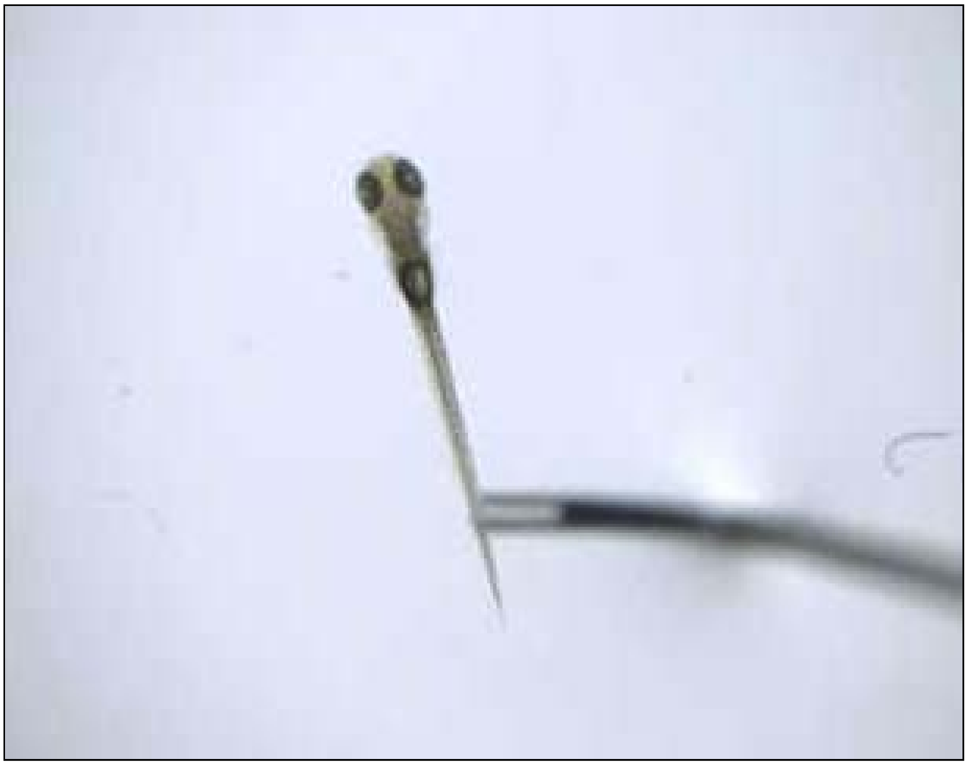

**Movie 1: Ctrl-MO injected larvae present normal motility at 6 dpf.** ctrl-MO injected larva at 6 dpf has been tested with tactile stimuli response assay and presents normal motility swimming in the opposite direction of the stimulus.

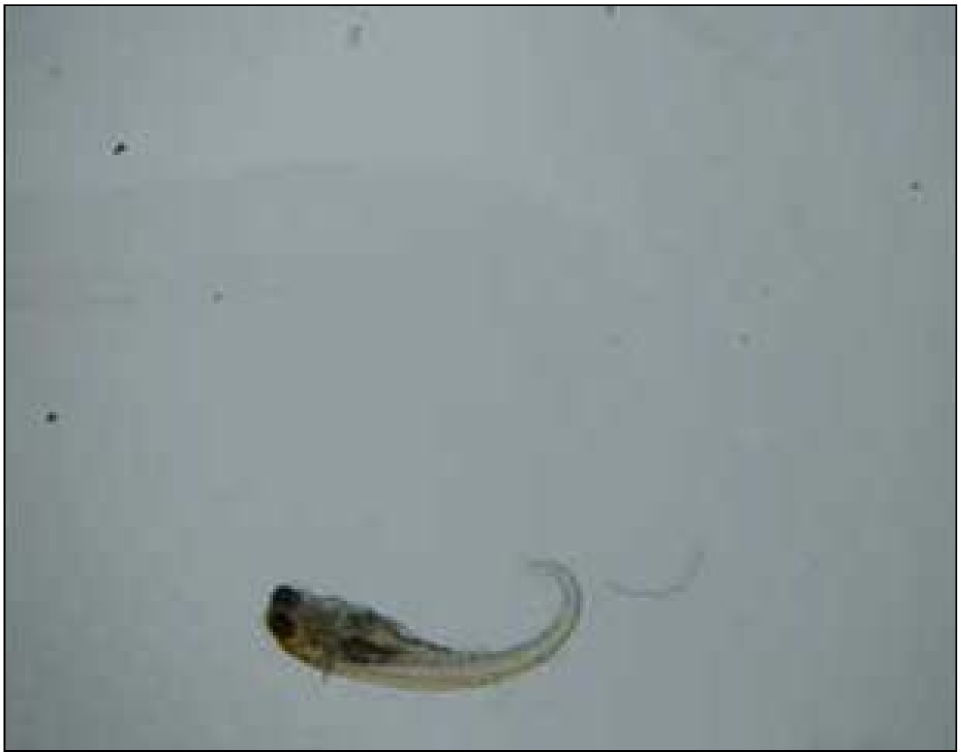

**Movie 2: *fas/fasl*-MO injected larvae present reduced motility at 6 dpf.** *fas/fasl*-MO injected larva, tested with a tactile stimulus response assay, is less sensitive to tactile stimuli than controls and does not swim away when touched.

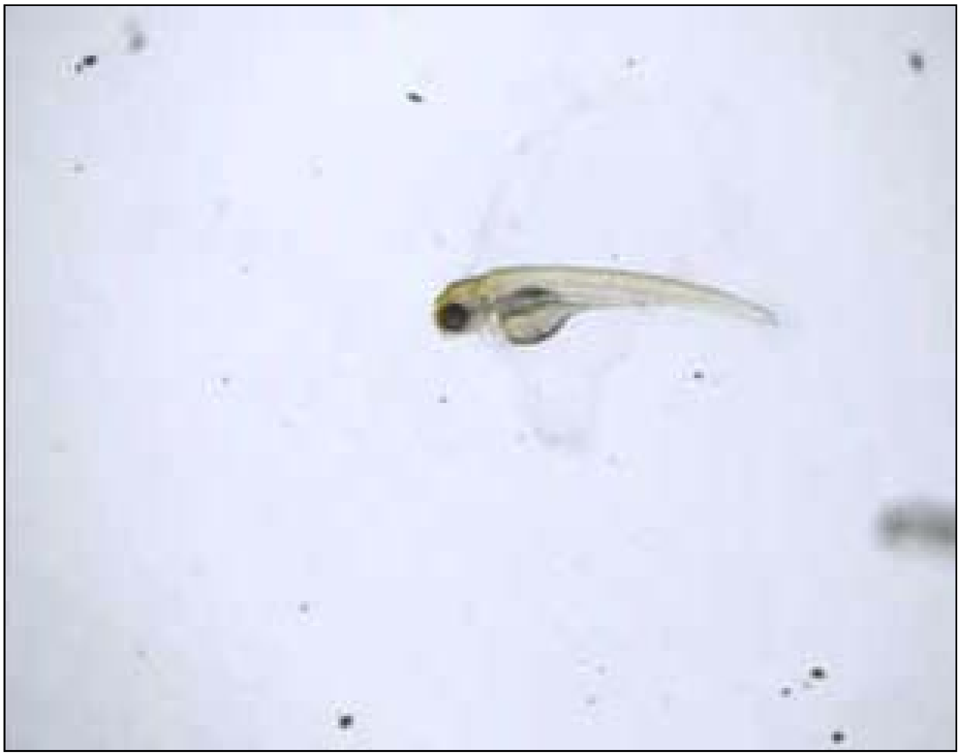

**Movie 3: Partial rescue of the motility of *fasl*-MO injected larvae by means of the *fasl* mRNA injection at 6 dpf.** Larva injected with *fasl*-MO and *fasl* full lenght mRNA partially rescues the immotile phenotype and swims away when stimulated.

**Supplementary Table S1. Primers sequences**

| Primer | Sequence |
|---|---|
| *FASL*_RTpcr_sense | 5’-GGCCTGTGTCTCCTTGTGAT-3’ |
| *FASL*_RTpcr_antisense | 5’-GCAGGTTGTTGCAAGATTGA-3’ |
| *T*_RTpcr_sense | 5’-CCAGCTCCAGTCAGTACC-3’ |
| *T*_RTpcr_antisense | 5’TACTGGCTGTCCACGATGTC |
| *fas*_RTpcr_sense | 5’-GTGACGCTAATGCAAAAATGAAG-3’ |
| *fas*_RTpcr_antisense | 5’-CGATGTCCTGCAGAGTGGTG-3’ |
| *fasl*_RTpcr_sense | 5’-CACTCGTCCCAACCAGTGTTC-3’ |
| *fasl*_RTpcr_antisense | 5’-CCACGCGAGAGTAAACGAAG-3’ |
| *beta-actin*_sense | 5’-TGTTTTCCCCTCCATTGTTGG-3’ |
| *beta-actin*_antisense | 5’-TTCTCCTTGATGTCACGGAC-3’ |
| *ntla*_qPCR_sense | 5’-CCTCGGGTTCGTACTGTGAG-3’ |
| *ntla*_qPCR_antisense | 5’-TCCGGAAGAGTTGTCCATGT-3’ |
| *col2a1a*_qPCR_sense | 5’-ATCCCATCATTTCACCTGGA-3’ |
| *col2a1a*_qPCR_antisense | 5’-TCTGTCCCTTTGCACCAAGT-3’ |
| *ef1alpha*_qPCR_sense | 5’-GGTACTTCTCAGGCTGACTGT-3’ |
| *ef1alpha*_qPCR_antisense | 5’-CAGACTTGACCTCAGTGGTTA-3’ |
| *fasl*-MO sense | 5’-gatcGATCGCAAAACATGAGTGCTAACTTCGGCC-3’ |
| *fasl*-MO antisense | 5’-gatcGGCCGAAGTTAGCACTCATGTTTGC-3’ |
| *fas*-MO sense | 5’-gatcTCTAATTCGGGTGACCTCCTCGA- 3’ |
| *fas*-MO antisense | 5’-gatcTCGAGGAGGTCACCCGAAT-3’ |
| *fasl_full_lenght*_sense | 5’-TTTGAATTCCGCCACCATGAGTGCTAACTT-3’ |
| *fasl_full_lenght*_antisense | 5’-TTTGCTCTAGAGATCAGTGGATCTTAAAGA-3’ |
| *fasl exon 1*_sense | 5’-ATGAGTGCTAACTTCGGCCAC-3’ |
| *fasl exon 4*_antisense | 5’-AAGCTGGCAGATTGCATTG-3’ |
| *fas exon 1*_sense | 5’-ATGCCCACTTTGACTTATAGC-3’ |
| *fas exon 7*_antisense | 5’-GATGAAGCCTCGACAATGTTC-3’ |

**Supplementary Table S2.** Morpholino sequences

| Morpholino | Sequence |
|---|---|
| *fas*ATG-MO | 5'-TCGAGGAGGTCACCCGAATTAGA-3' |
| *fasl*ATG-MO | 5'-GGCCGAAGTTAGCACTCATGTTTGC-3' |
| *splice-fas*-MO | 5'-TCCTGTAATACACAAACACATGCAG-3' |
| *splice-fasl*-MO | 5'-TACATTCTGTAGGTCTTACCTGTGT-3' |

## REFERENCES

1. Santoriello C, Gennaro E, Anelli V, Distel M, Kelly A, Koster RW, Hurlstone A and Mione M. Kita driven expression of oncogenic HRAS leads to early onset and highly penetrant melanoma in zebrafish. PLoS One. 5(12):e15170.
2. Distel M, Wullimann MF and Koster RW. Optimized Gal4 genetics for permanent gene expression mapping in zebrafish. Proc Natl Acad Sci U S A. 2009; 106(32):13365–13370.
3. Dale RM and Topczewski J. Identification of an evolutionarily conserved regulatory element of the zebrafish col2a1a gene. Dev Biol. 357(2):518–531.
4. Nasevicius A and Ekker SC.Effective targeted gene 'knockdown' in zebrafish.Nature genetics. 2000; 26(2):216–220.
5. Robu ME, Larson JD, Nasevicius A, Beiraghi S, Brenner C, Farber SA and Ekker SC. p53 activation by knockdown technologies. PLoS Genet. 2007; 3(5):e78.
6. Brusegan C, Pistocchi A, Frassine A, Della Noce.I, Schepis F, Cotelli F. Ccdc80-l1 Is involved in axon pathfinding of zebrafish motoneurons. PLoS One. 7(2):e31851.
